# Applicability of the Guide for Monitoring Child Development as a Telehealth Delivered Intervention During the Pandemic

**DOI:** 10.3389/fped.2022.884779

**Published:** 2022-06-03

**Authors:** Ezgi Ozalp Akin, Aysen Akbas, Sidika Canan Atasoy, Merve Cicek Kanatli, Selin Ince Acici, Revan Mustafayev, Bedriye Tugba Karaaslan, Hilmi Deniz Ertem, Bahar Bingoler Pekcici, Ilgi Ertem

**Affiliations:** ^1^Developmental Pediatrics Division, Department of Pediatrics, Ankara University School of Medicine, Ankara, Turkey; ^2^Department of Pediatrics, Acibadem Maslak Hospital, Istanbul, Turkey; ^3^Department of Child Development, Faculty of Health Sciences, Izmir Katip Çelebi University, Izmir, Turkey

**Keywords:** early childhood development, COVID-19, developmental difficulties, telehealth, early intervention, video observation, Guide for Monitoring Child Development (GMCD)

## Abstract

**Background:**

Early intervention delivered through telehealth is critically needed during crises, particularly for children in low and middle-income countries (LMICs). We aimed to determine the applicability of the international Guide for Monitoring Child Development (GMCD) intervention delivered through telehealth during the COVID-19 lockdown in Turkey.

**Methods:**

Using a mixed-methods longitudinal design, we recruited children with developmental difficulties aged 0–42 months with an appointment during the first lockdown at Ankara University Developmental Pediatrics Division and seen face-to-face only once before. Developmental pediatricians applied the GMCD intervention during a single telephone call. As a novel intervention component, caregivers were asked to record and send back videos of the child's development when there were doubts about the child's functioning. Caregivers were called 1 year later by blinded independent researchers and a semi-structured interview on applicability was conducted. Applicability of the caregiver recorded video component of the intervention was assessed by a blinded observer using the GMCD Video Observation Tool.

**Results:**

Of 122 children that received the telehealth delivered GMCD intervention, 114 (93.4%) were included in the 1-year outcome study. Most were boys (51.8%); median age was 16.5 (IQR: 10.0–29.0) months, 51.0% had chronic health conditions, and 66.7% had developmental delay. All caregivers that received the intervention were mothers; 75.4% had at least high school education. The intervention was reported as applicable by 80.7% with high levels of satisfaction. On multivariate regression analysis, absence of chronic health related conditions was significantly associated with applicability (OR = 2.87, 95% CI = 1.02–8.09). Of 31 caregivers that were asked for videos, 19 sent back 93 videos that were technically observable. One or more developmental domains were observed in all videos; in 52.6%, caregivers provided early learning opportunities.

**Conclusions:**

The findings of this study imply that the telehealth delivered GMCD intervention for children with developmental difficulties is applicable during the pandemic. The intervention content and frequency needs to be augmented for children with chronic health conditions. Further research is required to examine applicability and effectiveness of the GMCD intervention in other settings, particularly in LMICs.

## Introduction

Early intervention models for children with developmental difficulties that can be provided from a distance have emerged as a critical need during the coronavirus disease 2019 (COVID-19) pandemic. Developmental difficulties (DDs) are defined by the World Health Organization (WHO) and UNICEF as “any condition that puts a child at risk of suboptimal development, or that causes a child to have a developmental deviance, delay, disorder or disability” (1). For children with DDs strong evidence exists for the lifelong benefits of early intervention such as increased ability to learn, greater achievement in school and later life, participation in life, citizenship, and overall quality of life (2). Studies in the past 2 years have shown that face-to-face services for children with DDs have been negatively influenced by the COVID-19 pandemic in many countries, especially during the lockdowns (3–5). The unexpected and sudden decrease in early intervention services has compromised not only opportunities to support children's development during the early critical years of brain growth (2, 6) but also has added to caregiver social isolation, stress, burden and burn out (6–8), compromising the mental health (6–9) and well-being (10) of children and families.

Recent publications imply that during the pandemic, high-income countries have implemented early intervention services through telehealth to overcome the challenges of service delivery (11–14). Studies have already reported positive effects of telehealth delivered interventions for young children with DDs including acceptability (14–16), feasibility (14, 17, 18), family satisfaction (11, 19), improved child development (20) and behavior (14, 20, 21). Research on interventions delivered through telehealth for children with DDs, however, has been limited in three important aspects. First, sample sizes have been either small (<10) (17–19), or have included older children (11, 14, 15), hindering generalizability for the actual recipients of early intervention, infants and young children. Second, samples of studies have been restricted to one type of disorder such as autism spectrum disorder (14–16, 20) or Fragile X (21), limiting generalizability for young children with other common DDs. Third, almost all studies have been reported from high-income countries. Although some effective interventions exist, there is limited evidence on real-life applicability in clinical practice even in high-income countries (12).

Disparities and barriers to telehealth exist particularly between high-income and low and middle-income countries (LMICs) (22, 23) where most of the world population of children reside (24). We identified only one study from LMICs on early intervention delivered through telehealth. In this mixed-methods study from India, Sengupta et al. investigated the perceptions of parents about the online delivery of a parent-mediated autism spectrum disorder intervention for a dozen children aged 1–6 years and found the intervention to be beneficial and acceptable (16). The paucity of research on applicable telehealth interventions for young children with a range of difficulties from LMICs, calls for urgent reports on such interventions that can be used during the pandemic and thereafter.

The international Guide for Monitoring Child Development (GMCD) is a comprehensive package that enables monitoring and supporting child development, early identification of developmental risks and delays and early intervention for children aged 0–42 months. The monitoring development component of the GMCD has been standardized and validated in research conducted in four diverse countries Argentina, India, South Africa and Turkey and is applicable internationally without the need for restandardization and validation (25, 26). Two independent reviews, 5 years apart, have rated the GMCD monitoring component as the highest performing early development tool for disseminated use in LMICs (27, 28). Clinicians from over 30 countries have been trained on the GMCD and training of trainers has been completed in 13 countries. Research on the early intervention component of the GMCD has been reported from Turkey (29), India (30), Azerbaijan, Turkmenistan, and Kyrgyzstan (31) and a multi-country cluster-randomized trial of its effectiveness and cost-effectiveness is ongoing (32). The GMCD intervention has been used empirically for many years *via* telephone calls to provide distant services to children with DDs but its applicability as a telehealth delivered intervention has not been studied.

We had the opportunity to examine the applicability of the telehealth delivered GMCD intervention during the initial 3-month COVID-19 lockdown in Turkey. Turkey has universal health insurance coverage, and children with special health care or developmental needs are eligible for government-subsidized health, special education, center-based early intervention, rehabilitation, and social services. During the lockdown, all non-urgent health and other services were suspended, and preschools, daycare centers, special education, rehabilitation centers were closed. Ankara University Developmental Pediatrics Division (AUDPD) clinic continued to provide monitoring, support and early intervention for young children using the GMCD *via* telehealth. In this study, we aimed to examine the applicability by caregivers of the telehealth delivered GMCD intervention during the pandemic for young children with DDs served at AUDPD.

## Materials and Methods

### Study Design and Participants

We used a mixed-methods longitudinal study design and recruited children with developmental difficulties aged 0–42 months who had an appointment to be seen during the first lockdown in Turkey (March 17th to June 1st, 2020). To eliminate caregivers that may be motivated to report the GMCD intervention as “applicable” because of their prior multiple face-to-face clinical encounters, in this study we included children who had been seen face-to-face at AUDPD only once before the lockdown.

### Procedures

The GMCD intervention was adapted by its developer (IE) and the AUDPD team so that it could be delivered using telehealth. Three faculty members of AUDPD (EOA, BBP, IE) trained and provided supervision for four developmental pediatric fellows (AA, MCK, SCA, SIA). These clinicians delivered the GMCD intervention as the routine clinical service provided at AUDPD during the lockdown period. Eligible families were called up to five times and were categorized as “non-respondents” if the caregiver did not respond to any of these calls. The clinician administered the GMCD intervention during a single telephone call lasting ~40 min. First, the GMCD monitoring component was administered. Information on how the child was functioning in all of the developmental domains, strengths and delays in development as well as psychosocial strengths and risk factors were identified. Next, recommendations on how to support the child's development based on the child's individual functioning and strengths were discussed and a mutually developed plan was made with the caregiver on how to address risks factors and needs, employing a strengths-based approach. Face-to-face assessment of the child and demonstrations of how to support development were not possible during the telephone call. For cases in which the clinician or the caregiver had doubts regarding the child's functioning or how to promote the child's development, therefore, the clinicians asked the caregivers to record short relevant videos and to send these back *via* digital messaging through Whatsapp.

**Figure 1:**
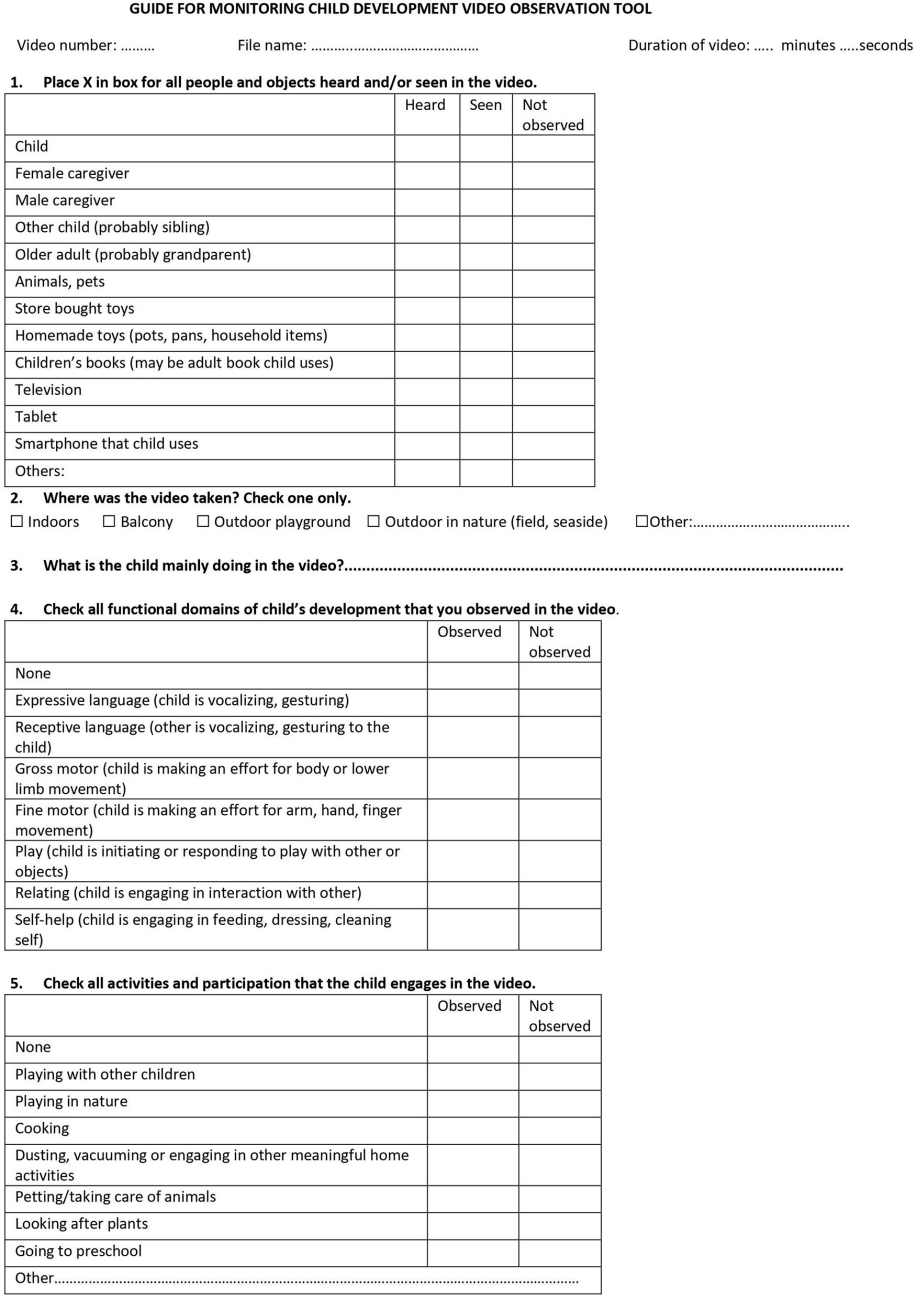
Item examples of the Guide for Monitoring Child Development Video Observation Tool.

To determine applicability, caregivers were contacted by telephone 1 year after the telehealth intervention by two independent researchers (BTK and RM) who were not Ankara University staff and who were blinded to the study aims and hypotheses. The researcher obtained oral consent (this was supplemented with written consent when families came back for clinic visits) and conducted a semi-structured interview on applicability lasting ~20 min. To determine the applicability of the parent recorded video component of the intervention, videos that were taken and sent back by the caregivers were evaluated using the GMCD Video Observation Tool (Figure 1). A researcher (HDE) blinded to the study aims and hypotheses as well as the content of the GMCD intervention obtained 95% reliability with the developer of the tool (IE) on 20 consecutive videos and coded all components of the tool for all videos.

### Intervention and Measures

*The Guide for Monitoring Child Development (GMCD) intervention* is a theory and research-based comprehensive package that enhances the knowledge, skills and attitudes of its users to monitor and support early childhood development and to address developmental difficulties (25, 26, 33–36). Grounded in bioecological theory (37), family-centered and strengths-based approaches (38), the GMCD also encompasses the World Health Organization (WHO) International Classification of Functioning, Disability and Health (ICF) (39) and Nurturing Care frameworks (1). The GMCD users can range in background from highly educated specialists (e.g., developmental pediatricians, pediatricians, early intervention specialists, and psychologists) to much less educated community health workers (e.g., in rural LMIC settings). The GMCD is unique as an intervention in that: (1) it can be applied universally and is inclusive encompassing all children with and without DDs rather than being disability specific; (2) its recommendations are individualized rather than being generic or age-specific; (3) it is co-creating in partnership with the family rather than being didactic; (4) it is comprehensive rather than being domain specific; (5) it is culturally responsive and adaptable rather than imposing culture specific recommendations.

The philosophy of the GMCD involves partnering with caregivers in watching, enjoying, and supporting development, witnessing, mirroring and enhancing strengths of children, caregivers and communities in supporting development and addressing DDs using strengths-based approaches. This philosophy is put into practice by enhancing the user's skills in asking open-ended questions and actively listening and observing with genuine interest, respect and compassion to identify, understand and celebrate strengths in the child, family and community. The open-ended GMCD questions elicit: (a) caregivers' narrative on the child's development in seven domains (expressive and receptive language, gross and fine motor, relating, play and self-help); (b) caregivers' concerns about the child's development; (c) child, family, and community strengths; (d) how the child's development is supported including nurturing, responsive care and early learning opportunities; (e) health related and/or psychosocial risk factors that may impede development; (f) specific needs of the child and/or family. The conversational technique of the GMCD facilitates establishment of a working relationship between the GMCD user and caregivers, acknowledges that caregivers are the experts on their child, fosters the family's confidence and creativity, and aims to empower families to deal with risks and vulnerabilities. The responses of the caregivers are coded into internationally standardized GMCD developmental milestones (25) providing a standardized assessment of the child's functioning, activities, and participation, and enabling validated identification of developmental delay (26). The individualized early intervention is seamlessly built on this information together with the family and is based on the child, family and community strengths, preferences, priorities, dreams and wishes; and aims to address specific needs, vulnerabilities. Furthermore, the GMCD intervention informs caregivers about early brain development and plasticity, the importance of supporting development, planning activities and participation at home, in the community, with a special emphasis on children's interaction with nature. The intervention is finalized by making a follow-up plan together with the family and referrals to available resources when necessary. The GMCD intervention particularly when used at a distance includes requesting caregivers to record and share with their clinician, short video clips of their child's functioning, activities and participation and how they promote development. Sharing information around caregiver recorded videos aims to strengthen the therapeutic bond between the clinician and the family by reminding caregivers that they are being “held in the mind” of their clinician. This approach also aims to enable clinicians to observe the child's functioning in their own environment, remind caregivers to promote development, enhance caregiver creativity, and provide unique opportunities for mutual discussion of individualized intervention plans instituted in the child's home and/or daily environment. The video sharing technique is regarded as a key component of the intervention delivered through telehealth and thus its applicability was examined in this study.

*GMCD Applicability Questionnaire* was developed by the AUDPD team for the purposes of this study. The tool comprises “entry” questions about how the family was during the pandemic year, followed by and semi-structured and open-ended questions on the caregiver perceived applicability of the GMCD intervention. Semi-structured questions were on comprehensibility, partnership, perceived effectiveness, adaptability and satisfaction (rated on a Likert scale from extremely dissatisfied to extremely satisfied). Examples of such questions and the responses of the caregivers are shown in **Table 2**. Open-ended questions pertained to what caregivers appreciated regarding the intervention and their recommendations on how to improve the GMCD telehealth intervention.

*GMCD Video Observation Tool* was developed for purposes of this study by the senior author and developer of the GMCD (IE). The aim of the tool is to determine whether caregivers understand and record on video the content that is requested of them during the GMCD intervention. Each of the following items of this observational tool is scored as “observed” or “not observed”: developmental functioning of the child in the GMCD domains; activities and participation of the child (being in nature, playing with other children, engaging in daily family life activities such as cooking, community events); and whether the caregivers' are engaged in promoting the child's development. The affective state of the child is also recorded. Examples of the tool items are shown in Figure 1.

### Data Analysis

We used descriptive statistics including frequencies for categorical data; means and standard deviations for normal distributions; and medians and interquartile ranges, otherwise. The Shapiro-Wilk test was used to test normal distributions. We used thematic qualitative analysis to determine caregiver reasons for satisfaction with the GMCD intervention and their suggestions for improvement. The primary outcome measure, “applicability” was dichotomized and defined as “applicable” if the caregiver: (a) reported remembering the telehealth intervention; (b) listed the intervention recommendations about how to support the child's development; and (c) reported having implemented most of these recommendations. If one or more of these conditions were not met, the intervention was defined as “inapplicable.” Based on empirical evidence, we hypothesized that child and family related factors that may increase caregiver focus on the child's development would be associated with “applicability.” Child and family related factors dichotomized and examined were sex, age (≤12 months vs. older), chronic health condition (absence vs. presence), maternal and paternal education (<high school vs. higher), number of children in family (single vs. multiple), and family constitution (nuclear vs. extended). We conducted bivariate analyses for associations between “applicability” and child and family related factors using the Pearson's chi-squared test or the Fisher's exact test where appropriate. Next, multivariate logistic regression analysis was applied entering the variables with *p*-values < 0.10 into a model to determine independent factors associated with “applicability”. For statistical significance 95% confidence intervals (CIs) were used. Statistical analyses were done using IBM SPSS 20.0 (SPSS Inc., Chicago, IL, USA) package program.

## Results

During the study period 468 children needed to be seen at AUDPD. Of these 227 (48.5%) had been seen more than once before the pandemic, 92 (19.6%) were older than 42 months, and 14 (2.9%) called to be seen for the first time and thus 333 were excluded from the study. Of the remaining eligible 135 children, 122 (90.3%) could be reached by telephone and received the telehealth delivered GMCD intervention. One year after the GMCD intervention, caregivers of 114 (93.4%) children could be reached and all provided consent for the study; eight families could not be reached.

### Sociodemographic, Health, and Developmental Characteristics

The sociodemographic characteristics of the sample are shown in Table 1. Most children were boys (51.8%); median age was 16.5 (IQR: 10.0–29.0) months. Approximately half (51.0%) of the children had chronic health conditions and 28.9% were followed for preterm birth history only. Children with chronic health conditions included those with chronic illness such as children such as renal failure, hepatic failure, congenital heart disease, leukemia, epilepsy, immune deficiency (31.6%); disabilities such as cerebral palsy, autism spectrum disorder (11.4%); and genetic syndromes such as children including Down syndrome, Klinefelter syndrome and DiGeorge syndrome 7.9%. Most children (66.7%) in the sample and 89.7% of children with chronic health conditions had developmental delay on at least one domain of the GMCD conducted during the telehealth intervention. All caregivers who received the intervention were mothers, most had at least high school education (75.4%) and were homemakers (73.7%). Most families had more than one child (65.8%). Fifteen (13.2%) families were residing outside of Ankara. In the 1-year period, COVID-19 infection occurred in nearly half (46.5%) of the households, children had not been infected with COVID-19 nor lost a family member. During the 1-year period after the GMCD intervention 103 (90.4%) children had received at least one face to face or telehealth follow up session at AUDPD. The median number of face-to-face visits was 2 (IQR: 1–2, range: 1–4), and telehealth visits was 1 (IQR: 1–2, range: 1–4). The remaining 11 (9.6%) children did not receive any other contact; 6 (5.3%) were followed in other centers that were closer in distance to their homes and 5 (4.4%) children lost the follow up.

**Table 1:** Sociodemographic, health, and developmental characteristics (*N* = 114).

	***n***	**%**
**Sex**
Boys	59	51.8
Girls	55	48.2
**Child age (months)**
0–12	40	35.1
13–24	34	29.8
25–42	40	35.1
Reasons for follow-up at AUDPD^*^
Chronic health conditions	58	51.0
Preterm birth history only	33	28.9
Isolated language delay	17	14.9
Isolated behavioral problems	6	5.2
Developmental delay on GMCD^**^domains
At least one domain	76	66.7
Expressive language	41	36.0
Receptive language	17	14.9
Gross motor	19	16.7
Fine motor	19	16.7
Relating	17	14.9
Play	18	15.8
**Maternal age (years)**
<20	1	0.9
20–30	46	40.4
31–40	55	48.2
>40	12	10.5
**Paternal age (years)**
<20	0	0.0
20–30	14	12.3
31–40	84	73.7
>40	16	14.0
**Maternal education**
Primary school or less	10	8.8
Secondary school	18	15.8
High school	34	29.8
University education or higher	52	45.6
**Paternal education**
Primary school or less	9	7.9
Secondary school	7	6.1
High school	35	30.7
University education or higher	63	55.3
**Maternal working status**
Homemaker	84	73.7
Employed	30	26.3

* *AUDPD, Ankara University Developmental Pediatrics Division*.

** *GMCD, International Guide for Monitoring Child Development*.

### Applicability of the GMCD Intervention Delivered Through Telehealth

Responses of caregivers to the *GMCD Applicability Questionnaire* are shown in Table 2. One year after the single telehealth intervention, all but one caregiver remembered being delivered the telehealth intervention and most (80.3%) stated that during the lockdown they had not received distant services from any sources other than AUDPD. Most caregivers (79.8%) reported remembering the name of the clinician who delivered the intervention; almost all (98.2%) remembered the intervention content. On the Likert scale, high levels of satisfaction were reported the intervention (median 10, IQR: 8–10). Examples of caregiver responses to the open-ended questions are also shown in Table 2. Qualitative analysis revealed two main themes related to caregivers' reasons for satisfaction with the intervention. These were (a) receiving practical information on how to support their child's development; (b) perceiving the call as “being held in their clinician's mind” which was reported as “being valued” and psychological support. Parents suggested that the intervention could be improved by increasing telehealth frequency and using video calls when possible.

**Table 2:** Examples from GMCD Applicability Questionnaire (*N* = 114).

**Examples of questions**	**Positive response**	**%**
Were you contacted by the AUDPD during the first lockdown?	113	99.1
What was the name of the clinician who contacted you?	91	79.8
What did the clinician recommend for your child and family? Can you list all that you remember?	112	98.2
Were the recommendations tailored for your child and family needs? Can you give examples?	110	96.5
Did the clinician give you opportunities to express yourself during the call? How?	108	94.7
Was the call useful for your child? In what way?	106	93.0
Did the call result in a positive difference for your child or family? How?	103	90.4
Were you able to implement the recommendations in your daily life? How?	92	80.7
**Examples of caregiver responses to open-ended questions**
“We felt so valued, even ….. (name of child) remembers her doctor.”		
“I got very pleased, I told my relatives and they were also surprised that my clinician called me.”		
“They cared for us, their suggestions were appropriate for our situation, we don't have enough money for toys or things like that, but we were given suitable suggestions.”		
“These suggestions were of great benefit for my daughter's development speech and walking.”		
“My clinician didn't force us to do anything. The way she talked and her closeness felt very good, she treated me like a sister or a mother.”		
“…(intervention) made a huge difference, there have been great changes in a good way about my daughter's daily life, about her behavior. Those suggestions were suitable to us, they were easy to apply with things that everyone has and can do at home.”		

The majority of mothers (80.7%) reported that the intervention was “applicable.” Table 3 shows odds ratios of child and family related factors associated with applicability. On bivariate analyses, absence of chronic health conditions (OR = 3.17, 95% CI = 1.14–8.85) was significantly associated with applicability. When child age (*p* < 0.10) was included in the logistic regression model, absence of chronic health conditions remained significantly associated with applicability (OR = 2.87, 95% CI = 1.02–8.09).

**Table 3:** Factors associated with applicability of telehealth delivered GMCD intervention (*N* = 114).

**Bivariate analyses**		**Proportions**		**GMCD intervention “applicable”**				
		***n***	**%**	***n***	**%**	**OR**	**95% CI**	***p***
**Child related factors**								
Sex	Girls	55	48.2	46	83.6	1.45	0.56–3.70	0.443
	Boys	59	51.8	46	78.0			
Age	≤ 12 months	40	35.1	36	90.0	2.89	0.91–9.24	0.064
	>12 months	74	64.9	56	75.7			
Chronic health	Absent	56	49.1	50	89.3	3.17	1.14–8.85	0.022
related condition	Present	58	50.9	42	72.4			
**Family and environment related factors**								
Maternal education	≥High school	86	75.4	72	83.7	2.06	0.76–5.59	0.152
	< High school	28	24.6	20	71.4			
Paternal education	≥High school	98	86.0	81	82.7	2.17	0.67–7.04	0.191
	< High school	16	14.0	11	68.8			
Number of siblings	Single child	39	34.2	32	82.1	1.14	0.42–3.10	0.792
	Has siblings	75	65.8	60	80.0			
Family structure	Nuclear	100	87.7	81	81.1	1.16	0.29–4.58	0.829
	Extended	14	12.3	11	78.6			
**Multivariate logistic regression analysis**								
Age ≤ 12 months						2.52	0.77–8.19	0.397
Absence of chronic health condition						2.87	1.02–8.09	0.047

Video recordings had been requested from 31 caregivers and 24 of these caregivers sent back a total of 109 videos. Sixteen videos could not be assessed due to technical reasons, leaving 93 videos of 19 children (61.3% of those asked to send videos) to be scored on the *GMCD Video Observation Tool*. The median number of videos sent back for each child was 3 (IQR: 2.0–5.8). The median duration of videos was 53 (IQR: 29–97) s. At least one GMCD developmental domain was observed in all of the videos. In most videos, gross motor (81.7%), fine motor (78.4%), expressive and receptive language (55.9%) domains were observed. Play and relating domains were observed in 44.1% and self-help domain was observed in 12.9% of the videos. In 46 (49.5%) videos a female caregiver, in 8 (8.6%) a male caregiver and in 6 (6.5%) a sibling was also observed apart from the child. In 49 (52.6%) videos parents were engaged in early learning opportunities for their children. Only 21 (22.6%) videos included store bought toys and 8 (8.6%) included homemade toys, 6 (6.5%) children's books. In only 7 (7.5%) videos children were engaged in activities and participation.

## Discussion

This study has provided information on the applicability of a single telehealth delivered GMCD intervention during the pandemic lockdown in Turkey. Most caregivers of young children with DDs followed at a developmental pediatrics clinic, remembered the intervention content, reported high levels of satisfaction, and had incorporated most of the intervention recommendations into their daily lives. Applicability of the GMCD intervention was similar across child sexes, ages, parental education levels and family constitutions. Families of children without chronic health conditions were ~3 times more likely to report the intervention as applicable. Our findings indicate that the GMCD intervention has the potential to be applied through telehealth and may help address the need for such early intervention models in LMICs.

There is a paucity of research on telehealth delivered intervention programs, specifically for children in their early years. Two studies from the United States have assessed parental satisfaction with such services (11, 12). In the study on 207 children with a variety of disabilities, parental satisfaction with face-to-face combined with telehealth services was examined during the pandemic. In response to the question “What is your overall level of satisfaction with the therapeutic services your child has received during the coronavirus pandemic?” medium to high satisfaction was reported by nearly half of the families (11). A smaller scale study on 17 children, similarly reported satisfaction with the telehealth intervention (12). In the only study identified from LMICs, parents of 12 children with autism spectrum disorder found the telehealth intervention beneficial and acceptable (16). The results of these studies are parallel to our findings and indicate that early intervention applied using telehealth during crises is acceptable and satisfactory for families of children with DDs in different cultures. Our study adds to the literature by applying an internationally standardized package for young children with a range of developmental difficulties and by obtaining longer term (1 year) information.

The COVID-19 pandemic has been detrimental to services for children with DDs (40). It has been reported that disparities in services have increased during the pandemic for children with DDs in LMICs (3). A study from the United States reported that during the pandemic, 72.0% of children with disabilities received video-based telehealth interventions (11) whereas only 19.7% of children in our study received different types of distant service outside of AUDPD apart from our intervention in the lockdown. Other than limited resources for service delivery in LMICs, barriers exist to telehealth interventions delivered using the internet and computers such as teleconference and video calls. Based on our empirical information, families express that the use of the camera increases internet use by their smartphones and at times it is not affordable for them. Furthermore, internet connection may be problematic. Poor internet connectivity (16, 41, 42), lack of telehealth infrastructure (42, 43), limited knowledge about how to use technology (42, 43), high electricity fares and frequent failures (4) have also been reported in the literature. Telephone calls therefore, appear to be the main route of telehealth at scale but studies on early intervention packages delivered through telephone calls have not been previously reported from LMICs.

In our study, caregivers of children without chronic health conditions were significantly more likely to report the GMCD intervention as applicable, compared to caregivers of children with chronic conditions. Although 72.4% of caregivers of children with chronic health conditions also found the intervention as applicable this rate was 89.3% for children without chronic conditions. Medical care has been reported as one of the main concerns of parents of children with chronic health conditions during the pandemic (4, 40). Parallel to these reports families in our study may have focused more on their child's health care needs than developmental needs. Research exists on the increased distress of caregivers of youth with physical illness during the pandemic (44) but studies on the needs of young children with chronic health conditions and their families is lacking. Our findings imply that children with chronic health conditions are a vulnerable group and more than a single telehealth intervention is needed to support the development of such children. Caregivers of children with chronic health conditions may be overwhelmed with taking care of the medical needs of their children and may need many more intense or frequent contact to address their needs. Further research is needed on the specific needs of young children with chronic conditions and how to address these at a distance during crises.

The GMCD intervention offers a theory and evidence-based, practical method for monitoring child development, early identification of developmental difficulties and delivery of early intervention *via* telephone calls which may be the only route available during crises in low resource settings. Our study has shown that as well as the telephone delivered intervention, the parent recorded video component of the GMCD intervention was applicable. Our analyses of video content indicates that caregivers understood what the videos should contain, and also put time and effort into recording and sharing what was requested by their clinicians. Two prior studies involving parent recorded videos have been published both during the pandemic. In a study from Italy, parents of children at risk for autism were requested to record play videos and a high return rate was reported (45). The second study, from India, aimed to conduct the General Movements Assessment of 11 high risk infants using parent recorded videos and also reported a high rate of return (46). Our study adds to this literature highlighting that video requests made during a one-time telephone delivered intervention have high return with appropriate content. The parent recorded video component of the GMCD intervention may provide an innovative individualized approach to assessing and promoting children's development when face to face, home-based services are not readily possible. The importance of play with home-made toys and materials, reading books, activities and participation in daily life are emphasized during the GMCD intervention, but these were rarely observed in the videos. Based on this finding, we plan to augment the GMCD intervention with respect to activities and participation for young children.

The main strength of this study is the mixed-methods longitudinal design which obtained detailed information on applicability and long term outcome. The high follow-up rate after 1 year is another strength which provides generalizability. Furthermore, to address detection bias independent blinded researchers were used to assess outcomes in both the 1-year follow-up and video content analysis. Our study has important limitations. The reliance on caregiver report to examine applicability may cause reporting bias with socially desirable responses. The intervention was provided in a non-experimental design and was a part of routine clinical services. Most children (90.4%) who had received the intervention also received follow up visits. Therefore, it was not possible to tease out whether the follow up contacts effected reports of mothers regarding applicability. Furthermore, maternal and paternal education was high in our sample. This may limit the generalizability of our findings to other LMIC settings with lower parental education levels. Our limited sample size in a single, urban setting are other important limitations, and call for more generalizable, preferably multi-country studies on applicability followed by effectiveness studies.

## Conclusions

In low and middle-income countries, there is a pressing need for early intervention models that can be delivered at a distance. The international Guide for Monitoring Child Development intervention delivered by clinicians through a single telephone call during the pandemic lockdown in Turkey was regarded as applicable by caregivers of children with developmental difficulties. When interviewed 1 year after the intervention, most caregivers remembered the intervention, reported high levels of satisfaction, and that they applied the intervention in their daily lives. Having a chronic health condition was associated with significantly fewer reports of applicability, implying that intervention content and frequency should be augmented for such children. Asking caregivers to record and send back videos of their child's development is a novel technique and a promising addition to the intervention. Further research is needed to determine whether the telehealth delivered Guide for Monitoring Child Development intervention is applicable in other settings and whether it is effective in supporting the development of young children with DDs.

## Data Availability Statement

The raw data supporting the conclusions of this article will be made available by the authors, without undue reservation.

## Ethics Statement

The studies involving human participants were reviewed and approved by Ankara University School of Medicine Ethics Committee. Written informed consent to participate in this study was provided by the participants' legal guardian/next of kin.

## Author Contributions

EOA, BBP, and IE finalized study design and methodology, supervised study. EOA and IE drafted the manuscript. AA, SCA, MCK, and SIA delivered the intervention and conducted data analyses. RM and BTK conducted the 1-year interviews and supported analysis of longitudinal outcome data. HE conducted the video assessments. EOA, SCA, AA, and SIA analyzed quantitative. MCK analyzed qualitative data. All authors contributed to the conceptualization, design of the study, writing of the manuscript, approved the final manuscript as submitted, and agreed to be accountable for all aspects of the work.

## Funding

This study was conducted using Ankara University internal funding.

## Conflict of Interest

The authors declare that the research was conducted in the absence of any commercial or financial relationships that could be construed as a potential conflict of interest.

## Publisher's Note

All claims expressed in this article are solely those of the authors and do not necessarily represent those of their affiliated organizations, or those of the publisher, the editors and the reviewers. Any product that may be evaluated in this article, or claim that may be made by its manufacturer, is not guaranteed or endorsed by the publisher.
